# Hospital acquired *Clostridium difficile* infection in pediatric wards: a retrospective case–control study

**DOI:** 10.1186/s40064-016-3013-8

**Published:** 2016-08-11

**Authors:** Ayse Karaaslan, Ahmet Soysal, Nurhayat Yakut, Gulsen Akkoç, Sevliya Ocal Demir, Serkan Atıcı, Nurver Ulger Toprak, Guner Söyletir, Mustafa Bakır

**Affiliations:** 1 Division of Pediatric Infectious Diseases, Department of Pediatrics, Marmara University School of Medicine, Fevzi Çakmak Mahallesi, Mimar Sinan Caddesi No: 41 Üstkaynarca, Pendik, Istanbul, Turkey; 2 Department of Microbiology, Marmara University School of Medicine, Istanbul, Turkey

**Keywords:** *C. difficile* infection, Hospitalized children, Antibiotic use

## Abstract

**Background:**

*Clostridium difficile* is a major cause of antibiotic-associated diarrhea and frequently results in healthcare-associated infections. The aim of this study was to determine the incidence and potential risk factors for *C. difficile* infection (CDI) in hospitalized children who developed diarrhea. A retrospective study was performed at a university hospital in İstanbul over a three-year period (2012–2014).

**Results:**

During the study period 12,196 children were hospitalized, among them 986 (8 %) children with diarrhea were investigated for CDI and 100 (0.8 %) children were diagnosed with CDI. The incidence of CDI in hospitalized children was 4/1000, 9/1000 and 9/1000 patients per year in year 2012, 2013 and 2014, respectively (p = 0.008, p < 0.01). The mean age of children with CDI (2.6 ± 2.6 months) was lower than children without CDI (57.5 ± 63.5 months) [p = 0.001]. In the multivariate analysis, the presence of underlying chronic diseases [presence of malnutrition (OR 7, 95 % CI 1.33–36.7, p = 0.021), presence of solid organ tumors (OR 6, 95 % CI 2.4–15.7, p < 0.00), presence of congenital heart diseases (OR 4.6, 95 % CI 1.13–18.7, p = 0.03), hospitalization in PICU (OR 15.6, 95 % CI 3.2–75.8, p = 0.001) and hospitalization in hematology and oncology ward (OR 7.8, 95 % CI 2–29.9, p = 0.002)] were found to be independent risk factors for CDI.

**Conclusion:**

This is the first description of the incidence and associated risk factors of CDI in Turkish children. One of the most important risk factor was prior antibiotic exposure which emphasizes the importance of antibiotic stewardship programs.

## Background

*Clostridium difficile* is an anaerobic Gram positive, toxin-producing and spore forming bacillus that can cause a range of illnesses from antibiotic-associated mild diarrhea to fulminant diseases such as pseudomembranous colitis, sepsis and death (Nylund et al. 2011). *C. difficile* spores are resistant to heat, disinfectants and antibiotics and they can survive in hospital environment for several months. Contaminated medical devices lead to transmission to patients, especially by health care worker’s hands (Kim et al. 1981).

*Clostridium difficile* is one of the most common healthcare-associated infections and it is responsible for 15–25 % of cases of nosocomial antibiotic-associated diarrhea (Lessa et al. 2015; Bartlett 2002). *C. difficile* can be detected in stool specimens without symptoms in up to 70 % of children during the first year of life (Bryant and McDonald 2009). Colonization rates decrease as age increases and after almost 4 years of age becomes less than 5 % (Vernacchio et al. 2006). It causes diarrhea due to alteration of colonic flora, which is generally caused by antibiotic therapy and ingestion of the microorganism leading to toxin production followed by mucosal injury and inflammation (Nylund et al. 2011). *C. difficile* infection (CDI) is related to A and B toxins, which are the major virulence factors of *C. difficile.* They both cause mucosal damage; however, Toxin B is about ten times more virulent than Toxin A. CDI severity is associated with the fecal toxin levels (Lyras et al. 2009; Warny et al. 2005). There is also a hypervirulent strain of *C. difficile* (NAP1/BI/027) which is associated with more violent disease (Petrella et al. 2012).

CDI is more common in adults than children; however, recent studies reported that CDI hospitalization rates are increasing in children, especially as a result of increased use of antibiotics and changes in the demographics of hospitalized patients. 1–5-year old children have the highest incidence of CDI (Deshpande et al. 2013; Zilberberg et al. 2010; Khanna and Pardi 2012). The most important and well-known risk factor for CDI is antibiotic exposure, with penicillins, clindamycin, cephalosporins, and fluoroquinolones implicated in the majority of cases (Nylund et al. 2011; Leffler and Lamont 2015). Children exposed to proton pump inhibitors, acid suppression medication, and those who have a gastrostomy or jejunostomy tube are also at risk of CDI (Freedberg et al. 2015; Turco et al. 2010; Sandora et al. 2011).

This study was performed to report the incidence of *C. difficile* and its risk factors in hospitalized children in our hospital, as no previous data exists in our country.

## Methods

The study was performed in a tertiary university hospital with a 649-bed capacity including all major pediatric wards and neonatal and pediatric intensive care units. We retrospectively reviewed the medical records and ICD codes of pediatric patients aged between 0 months and 18 years who had the diagnosis of diarrhea and ICD (International Classification of Diseases) code for *C. difficile* between January 2012 and December 2014. Patients’ age and gender distribution, pediatric ward type, number of patients with underlying diseases, implicated antibiotics, and length of antibiotic usage were noted. The study protocol was approved by the Ethics Committee of Marmara University Medical School. Informed written consent was obtained from all participants’ parents/guardians on admission for all diagnostic tests and treatment protocol.

Patient group consisted of all children who developed diarrhea 48 h after hospitalization in whom CDI was diagnosed. Children with community aquired diarrhea were excluded from the study. A case–control study was conducted, comparing CDI patients with non-CDI patients who had diarrhea. The control group consisted of children who developed diarrhea 48 h after hospitalization in whom CDI was investigated, but toxin A and/or toxin B were negative for *C. difficile* in stool specimens.

CDI was defined as the presence of the following two findings: (1) diarrhea defined as three or more unformed stools within 24 h, (2) a positive cytotoxic stool assay for the presence of toxin A and/or toxin B (Cohen et al. 2010). Qualitative detection of toxins A and B of *C. difficile* was performed using Premier toxins A and B (*C. difficile*) EIA kit bioMérieux, (Marseille, France) according to the manufacturer’s instructions.

All patients with the diagnosis of health care associated CDI that had clinical symptoms such as fever and/or increase of white blood cell count received antimicrobial therapy. Metronidazole (30 mg/kg per day divided into 3 or 4 doses) was administered for a minimum of 10 days.

For statistical analysis, the Number Cruncher Statistical System (NCSS) 2007 (Kaysville, Utah, USA) statistical software programs was used. For univariate analysis, student’s test, Pearson χ^2^, and Fisher-Freeman-Halton exact tests were used for categorical and continuous variables. Categorical variables were further tested if appropriate in a multivariate analysis by using logistic regression analysis. Significance was set at p < 0.05.

## Results

Between January 2012 and December 2014, 12196 children were hospitalized in our hospital. Among them 986 (8 %) children who developed diarrhea 48 h after hospitalization were investigated for CDI. In total, 100 (0.8 %) children developed CDI. The incidence of CDI in hospitalized children was 4/1000, 9/1000, and 9/1000 patient per year in years 2012, 2013, and 2014, respectively (p = 0.008; p < 0.01). The mean age of children with CDI was 2.6 ± 2.6 months. The median time for hospitalization in CDI patients was 12 days (range 1–180, mean 24.2 ± 32.8). Among the 100 patients diagnosed with CDI, 69 (69 %) received antibiotic therapy prior to development of diarrhea; and among control patients 522 (58.9 %) received antibiotic therapy. The most common antibiotics used among total patients were meropenem (n = 158, 16 %), piperacillin–tazobactam (n = 122, 12.4 %), vancomycin (n = 121, 12.3 %), ceftriaxone (n = 95, 9.6 %), teicoplanin (n = 65, 6.6 %), cefepime (n = 61, 6.2 %), liposomal amphotericin b (n = 54, 5.5 %), ampicillin (n = 51, 5.2 %) and ampicillin-sulbactam (n = 50, 5.1 %).

Univariate analysis found that patients’ age, presence of underlying chronic diseases, hospitalization in pediatric intensive care unit (PICU), hospitalization in hematology/oncology ward, and antibiotic usage prior to development of diarrhea were risk factors (Table 1) for developing CDI.

**Table 1:** Univariate analysis of risk factors for *C. difficile* infection among hospitalized patients

Variable	CDI n, (%)	Non-CDI n, (%)	p value
Mean age (month)	2.62 ± 2.59	57.4 ± 63.57	0.001
Gender			
Female	37 (8.5)	397 (91.5)	0.136
Male	63 (11.4)	489 (88.6)	
Presence of underlying chronic diseases	83 (83)	636 (71.7)	0.004
Hematology/oncology stay	41 (11.8)	305 (88.2)	0.005
PICU stay	13 (17.1)	63 (82.9)	0.001
Antibiotic usage prior to CDI	69 (11.7)	522 (88.3)	0.051

*C. difficile*: *Clostridium difficile*, *CDI* *C. difficile* infection, *PICU* pediatric intensive care unit

The multivariate analysis found that the presence of underlying chronic diseases [presence of malnutrition (OR 7, 95 % CI 1.33–36.7, p = 0.021), presence of solid organ tumors (OR 6, 95 % CI 2.4–15.7, p < 0.00), presence of congenital heart diseases (OR 4.6, 95 % CI 1.13–18.7, p = 0.03), hospitalization in PICU (OR 15.6, 95 % CI 3.2–75.8, p = 0.001) and hospitalization in hematology and oncology ward (OR 7.8, 95 % CI 2–29.9, p = 0.002)] were independent risk factors (Table 2). Older age (OR 0.72, 95 % CI 0.6–0.79, p < 0.001) was found to be protective for CDI.

**Table 2:** Multivariate analysis of risk factors for *C. difficile* infection among hospitalized patients

Variable	CDI n, (%)	Non-CDI n, (%)	OR	95 % CI	p value
Presence of underlying chronic diseases	83 (11.5)	636 (88.5)	–	–	0.005
Malnutrition	5 (20)	20 (80)	7	1.3–36.7	0.021
Solid organ tumor	23 (15.8)	123 (84.2)	6.1	2.4–15.7	0.001
Congenital heart disease	7 (23.3)	23 (76.7)	4.5	1.1–18.6	0.033
Hematology/oncology stay	41 (11.8)	305 (88.2)	7.8	2–29.9	0.002
PICU stay	13 (17.1)	63 (82.9)	15.6	3.2–75.8	0.001

*C. difficile*: *Clostridium difficile*, *CDI* *C. difficile* infection, *PICU* pediatric intensive care unit

The total hospital costs included all costs associated with hospitalization. Although the total hospitalization cost of patients with CDI (total cost was $4578) were higher than those without CDI (total cost was $3528), this difference was not statistically significant (p = 0.169). Mean length of hospitalization in the CDI group was 24.2 ± 32.8 days, compared to 21.3 ± 24.9 days in the non-CDI patients with diarrhea. The length of hospitalization in CDI group was longer; however, the difference was not significant (p = 0.304). In our study, there were no children unresponsive to treatment and all 100 patients showed clinical remission with the clearence of the symptoms. No CDI related mortality was observed.

## Discussion

Our study found the incidence of CDI in hospitalized children to be 4/1000, 9/1000, and 9/1000 per year in 2012, 2013, and 2014, respectively and this is the first report of CDI incidence in children in Turkey. We are therefore unable to compare our study with any other study in Turkey. Zilberberg et al. (2010) reported that pediatric CDI hospitalization rates increased from 7.2 to 12.8 from 1997 through 2006. The anticipated incidence of CDI for children under 18 years of age was 24.2 cases per 100,000 population according to active surveillance in the United States in 2011 and suprisingly about two-thirds of the cases were community associated CID (Lessa et al. 2015). Many children with community associated CDI lack the classicial risk factor of antibiotic exposure (Tschudin-Sutter et al. 2013). One of the newer studies from United States showed that CDI incidence in hospitalized children increased from 24.0 to 58.0 per 10,000 discharges per year (Pant et al. 2016). We also found increasing incidence in our study, similar to the literature.

Newborns rarely develop symptomatic *C. difficile* infection because of the protective effect of maternal antibodies, premature immune system and maybe the lack of intestinal receptors for *C. difficile* toxins (Nylund et al. 2011; McFarland et al. 2000). In our study median age of the patients with CDI was 2.6 ± 2.5 months. In infant population, three or more stools per day can be accepted as normal physiology. For this reason, diarrhea was accepted as at least three or more watery stools that exceeds the child’s usual number of daily stools, and the diagnosis of CDI was made by a neonatologist in all newborns. Colonization with *C. difficile* is common in infants; however, we did not screen for asymptomatic colonization, we only investigated toxins A and B of *C. difficile* in stool samples in children with diarrhea, fever, and/or increase of white blood cell count. Our hospital is a tertiary university hospital and critically ill patients are usually transferred to our hospital, explaining the reason for a low average age. Although we tried to diagnose CDI, we think we could not definetly distinguish infection from colonization because of the younger age of the study group. All patients were reported to the infection control committee and isolated in a private room. In addition to standard precautions, contact precautions were also taken for the duration of the illness.

Many studies have investigated the risk factors for CDI in hospitalized patients. Previous studies have reported that antibiotic exposure is the leading risk factor associated with CDI and proton pump inhibitors, acid suppression medication, gastrostomy or jejunostomy tubes, and malignancy have also been found as risk factors (Leffler and Lamont 2015; Freedberg et al. 2015; Turco et al. 2010; Sandora et al. 2011; Cohen et al. 2010; Samady et al. 2014). In our study, we found the presence of underlying chronic diseases such as malnutrition, congenital hearth diseases, malignancy, and stay in PICU as risk factors for CDI. We believe that malnutrition, admission to PICU, and malignancy may cause secondary immunocompromisation that facilitates CDI. However, congenital hearth disease was a suprising find as a risk factor for CDI, which was not previously defined. Although the total hospitalization costs of patients with CDI was higher than those without CDI, the difference was not statistically significant.

The treatment of CDI is based on discontinuation of inciting antibiotics, supportive care, and antimicribial therapy. Metronidazole is recommended for the therapy of mild or moderate CDI; however, vancomycin is preferred in severe or complicated diseases in children and adolescents for 10–14 days (Schutze and Willoughby 2013). In our study, we preferred metronidazole as the first agent, used for a minimum 10 days.

In our practical approach, we also investigate the hospitalized patients with diarrhea for rotavirus, adenovirus, giardia antigen, entamoeba antigen and bacterial stool culture which are possible to be diagnosed in our microbiological department. However because of our retrospective study design, in this study, we have only recorded the data of patients with the diagnosis of CDI whom were negative for other investigated pathogens.

This study has several limitations. Firstly, we did not collect all the ethiological agents of children with diarrhea without CDI. Secondly, we compared the risk factors in patients with diarrhea with CDI and children with diarrhea due to non-CDI; we did not compare patients with CDI between themselves. We compared the total hospital cost of patients with CDI and patients with diarrhea without CDI and the difference was not significant. However, if we had compared the patients with CDI and patients without diarrhea, we believe the difference would be significant, similar to previous reports in literature (McGlone et al. 2012).

## Conclusion

In this study, CDI was responsible for 10 % of cases of nosocomial-associated diarrhea. Prior antibiotic exposure was found to be a risk factor, emphasizing the importance of antibiotic stewardship programs. Additionally, PICU stay and hematology/oncology ward stay were the independent risk factors for CDI. These factors are uncontrollable and therefore infection control programs are the most important measures to prevent CDI.

## Abbreviations

*C. difficile*: *Clostridium difficile*
CDI: *Clostridium difficile* infection
ICD: International Classification of Diseases
PICU: pediatric intensive care unit
NCSS: Number Cruncher Statistical System

## References

[CR1] Bartlett JG (2002). Clinical practice. Antibiotic-associated diarrhea. N Engl J Med.

[CR2] Bryant K, McDonald LC (2009). *Clostridium difficile* infections in children. Pediatr Infect Dis J.

[CR3] Cohen SH, Gerding DN, Johnson S (2010). Clinical practice guidelines for *Clostridium difficile* infection in Adults: 2010 update by the Society for Healthcare Epidemiology of America (SHEA) and the Infectious Diseases Society of America (IDSA). İnf Control Hosp Epidemiol.

[CR4] Deshpande A, Pant C, Anderson MP (2013). *Clostridium difficile* infection in the hospitalized pediatric population: increasing trend in disease incidence. Pediatr Infect Dis J.

[CR5] Freedberg DE, Lamousé-Smith ES, Lightdale JR (2015). Use of acid suppression medication is associated with risk for *C. difficile* ınfection in infants and children: a population-based study. Clin Infect Dis.

[CR6] Khanna S, Pardi DS (2012). *Clostridium difficile* infection: new insights into management. Mayo Clin Proc.

[CR7] Kim KH, Fekety R, Batts DH (1981). Isolation of *Clostridium difficile* from the environment and contacts of patients with antibiotic-associated colitis. J Infect Dis.

[CR8] Leffler DA, Lamont JT (2015). *Clostridium difficile* infection. N Engl J Med.

[CR9] Lessa FC, Mu Y, Bamberg WM (2015). Burden of *Clostridium difficile* infection in the United States. N Engl J Med.

[CR10] Lyras D, O’Connor JR, Howarth PM (2009). Toxin B is essential for virulence of *Clostridium difficile*. Nature.

[CR11] McFarland LV, Brandmarker SA, Guandalini S (2000). Pediatric *Clostridium difficile:* a phantom menace or clinical reality?. J Pediatr Gastroenterol Nutr.

[CR12] McGlone SM, Bailey RR, Zimmer SM (2012). The economic burden of *Clostridium difficile*. Clin Microbiol Infect.

[CR13] Nylund CM, Goudie A, Garza JM (2011). Clostridium difficile infection in hospitalized children in the United States. Arch Pediatr Adolesc Med.

[CR14] Pant C, Deshpande A, Gilroy R, Olyaee M, Donskey CJ (2016). Rising incidence of clostridium difficile related discharges among hospitalized children in the United States. Infect Control Hosp Epidemiol.

[CR15] Petrella LA, Sambol SP, Cheknis A (2012). Decreased cure and increased recurrence rates for Clostridium difficile infection caused by the epidemic *C. difficile* BI strain. Clin Infect Dis.

[CR16] Samady W, Pong A, Fisher E (2014). Risk factors for the development of *Clostridium difficile* infection in hospitalized children. Curr Opin Pediatr.

[CR17] Sandora TJ, Fung M, Flaherty K (2011). Epidemiology and risk factors for *Clostridium difficile* infection in children. Pediatr Infect Dis J.

[CR18] Schutze GE, Willoughby RE (2013). Committee on Infectious Diseases, American Academy of Pediatrics. *Clostridium difficile* infection in infants and children. Pediatrics.

[CR19] Tschudin-Sutter S, Tamma PD, Naegeli AN (2013). Distinguishing community-associated from hospital-associated *Clostridium difficile* infections in children: implications for public health surveillance. Clin Infect Dis.

[CR20] Turco R, Martinelli M, Miele E (2010). Proton pump inhibitors as a risk factor for paediatric *Clostridium difficile* infection. Aliment Pharmacol Ther.

[CR21] Vernacchio L, Vezina RM, Mitchell AA (2006). Diarrhea in American infants and young children in the community setting: incidence, clinical presentation and microbiology. Pediatr Infect Dis J.

[CR22] Warny M, Pepin J, Fang A (2005). Toxin production by an emerging strain of *Clostridium difficile* associated with outbreaks of severe disease in North America and Europe. Lancet.

[CR23] Zilberberg MD, Tillotson GS, McDonald C (2010). *Clostridium difficile* infections among hospitalized children, United States, 1997-2006. Emerg Infect Dis.

